# Evaluation of gantry angle during respiratory‐gated VMAT using triggered kilovoltage x‐ray image

**DOI:** 10.1002/acm2.12683

**Published:** 2019-07-29

**Authors:** Ryohei Miyasaka, Hidetoshi Saitoh, Toru Kawachi, Tetsurou Katayose, SangYong Cho, Ryohei Yamauchi, Ryusuke Hara

**Affiliations:** ^1^ Department of Radiation Oncology Chiba Cancer Center 666‐2 Nitona‐chou, Chuo‐ku Chiba Japan; ^2^ Graduate School of Human Health Sciences Tokyo Metropolitan University 7‐2‐10 Higashiogu, Arakawa‐ku Tokyo Japan

**Keywords:** gantry angle, log analysis, quality assurance, respiratory‐gated VMAT, triggered image

## Abstract

Respiratory‐gated volumetric modulated arc therapy (gated VMAT) involves further complexities to the dose delivery process because the gantry rotation must repeatedly stop and restart according to the gating signals. In previous studies, the gantry rotation performances were evaluated by the difference between the plan and the machine log. However, several reports pointed out that log analysis does not sufficiently replicate the machine performance. In this report, a measurement‐based quality assurance of the relation between the gantry angle and gate‐on or gate‐off using triggered kilovoltage imaging and a cylinder phantom with 16 ball bearings is proposed. For the analysis, an in‐house program that estimates and corrects the phantom offset was developed. The gantry angle in static and gated arc delivery was compared between the machine log and the proposed method. The gantry was set every 5 deg through its full motion range in static delivery, and rotated at three speeds (2, 4 and 6 deg s^‐1^) with different gating intervals (1.5 or 3.0 s) in gated arc delivery. The mean and standard deviation of the angular differences between the log and the proposed method was −0.05 deg ± 0.12 deg in static delivery. The mean of the angular difference was within ±0.10 deg and the largest difference was 0.41 deg in gated arc delivery. The log records the output of the encoder so that miscalibration and mechanical sagging will be disregarded. However, the proposed method will help the users to detect the mechanical issues due to the repeated gantry stops and restarts in gated VMAT.

## 1 INTRODUCTION

The goal of radiotherapy is to deliver a prescribed dose to the target volume and to minimize side effects in normal tissue. In thoracic and abdominal regions, respiratory motion may be a major cause of geometrical uncertainty. As a technique to reduce this uncertainty, in respiratory gating radiotherapy, the target motion is tracked continuously and the radiation beam is delivered only in a specific range of the respiratory cycle.^1^, ^2^, ^3^ Recently, a technique combining respiratory gating and volumetric modulated arc therapy (gated VMAT) is applied for thoracic and abdominal tumors.^4^, ^5^, ^6^ Gated VMAT can deliver a dose to the target volume with high conformity despite the presence of respiratory motion.^7^

However, this technique involves further complexities to the dose delivery process because the gantry rotation must repeatedly stop and restart according to the gating signals. When respiration reaches the gate‐off phase, the radiation beam is turned off. However, the gantry slightly overruns from the intended stop position, and then the gantry is rewound to the restart position before the next gate‐on phase. When the gantry rotation speed is fast and/or the interval between beam‐off and beam‐on is short, the accuracy of the gantry angle may deteriorate. Regarding the accuracy of the gantry angle, the American Association of Physicists in Medicine Task Group 142 Report recommends ±1 deg as an acceptable criterion.^8^

In previous studies evaluating the machine performance of gated VMAT, the difference between the planned and the recorded gantry angle was analyzed using the machine log. Nicolini et al. reported that systematic delivery errors between the ungated and various gated conditions were not observable in the machine log (monitor unit, gantry angle or multileaf collimator positions). On the other hand, the gamma‐index passing rates between the planned and the measured dose distributions were changed within 2 % depending on the number of beam interruptions.^9^ Qian et al. found that the log‐based dose reconstruction faithfully realized the measurement of ion chamber array in gated VMAT under a variety of periodic respiration situations.^10^ The dose verification can evaluate machine performance comprehensively, however the mechanical issue due to the repeated stops and restarts of the gantry are not assured independently. Several reports discussed the log analysis and pointed out that log does not sufficiently replicate the machine performance.^11^, ^12^, ^13^ Therefore, measurement‐based quality assurance (QA) must be performed. A recent report on gantry angle analysis using radiochromic film concluded that the gantry angle error was smaller than 0.5 deg under a gantry rotation speed of 2.3 deg s^‐1^. Hubley et al. selected a slower gantry rotation speed so that the film‐based QA is achieved with a high signal‐to‐noise ratio.^14^ However, the gantry rotation speeds in gated VMAT vary irregularly from its maximum of 6.0 deg s^‐1^ to slower speeds. For gantry angular analysis under actual conditions, a more sensitive device than radiochromic film is required.

This report proposes a new method of using a kilovoltage (kV) imaging system and a cylinder phantom for measurement‐based QA of the gantry angle, and presents a comparison between measurement‐based QA and log analysis.

## 2 MATERIALS AND METHODS

### 2.A Experimental setup

**Figure 1:**
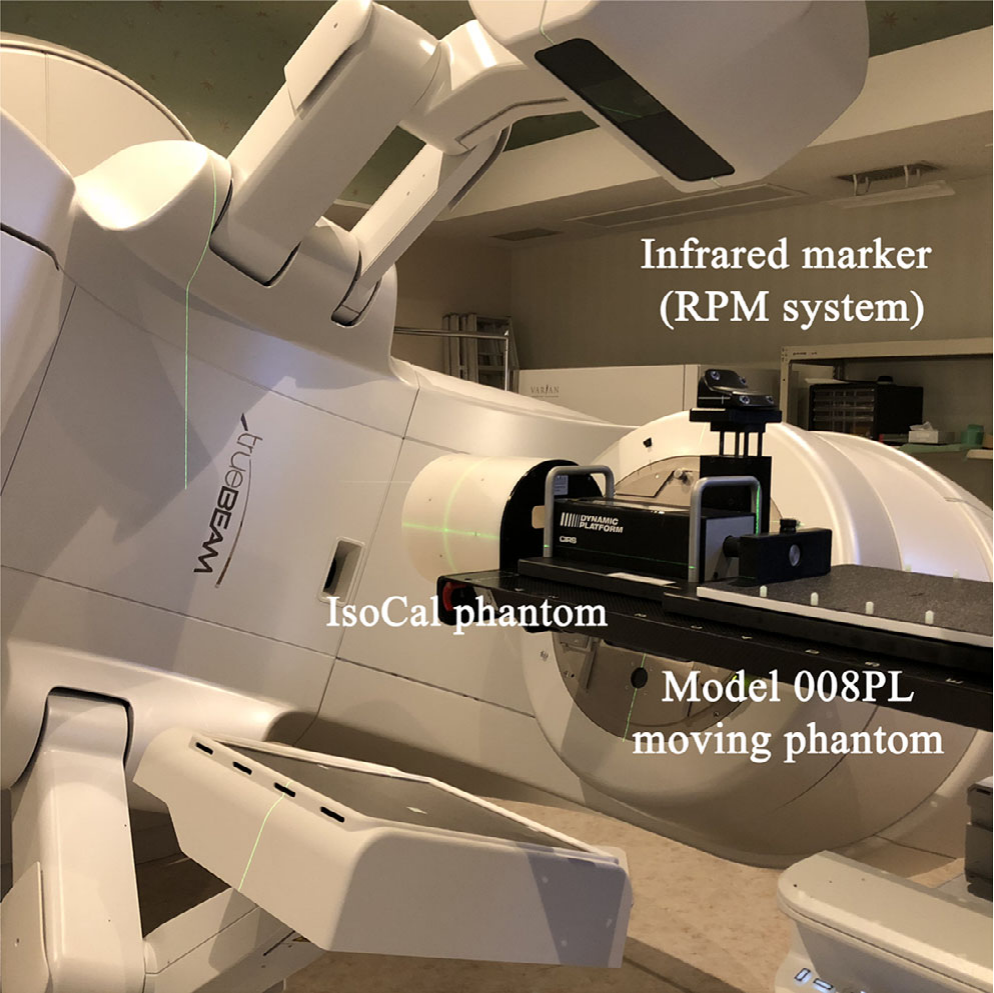
The experimental setup for the gantry angle analysis. The IsoCal phantom was mounted on the treatment couch and aligned with the room lasers. The gating signals were supplied to TrueBeam using the real‐time position management system and model 008PL moving phantom.

As shown in Fig. 1, a QA phantom (IsoCal, Varian Medical Systems, Palo Alto, CA, USA) was mounted on the tip of the linac (TrueBeam ver.2.5, Varian Medical Systems, Palo Alto, CA, USA) couch and aligned with the room lasers. The IsoCal phantom is a hollow cylinder with 16 tungsten‐carbide ball bearings (BBs) arranged at a specified position.^15^ While the purpose of this phantom is to calibrate between the isocenter and the image center,^16^ it was used to determine the gantry angle in this study. The phantom images were acquired by the “triggered imaging” mode using the on‐board kV imager at the source‐detector distance (SDD) of 150 cm. The active area is 40 cm × 30 cm and the pixel size is 0.39 mm in this imaging mode. The images were sampled at the moment of gate‐on or gate‐off in an exposure time of 50 ms. The gating signals were supplied to TrueBeam by the real‐time position management (RPM, Varian Medical Systems, Palo Alto, CA, USA) system and the moving phantom (008PL, CIRS, Norfolk, VA, USA) simulated the sinusoidal motion.

### 2.B Determination of the gantry angle

To determine the gantry angle, the projected coordinate of *i*th BB on the imager $\left(X_{i\;calc},\;Y_{i\;calc}\right)$ for given gantry angle θ and the source‐detector distance *Z* was calculated in advance. The coordinate of *i*th BB (*x_i_*, *y_i_*, *z_i_*) in the reference coordinate system defined by IEC61217 was transformed with the matrix for translation and rotation $M_{v}\left(\theta -\frac{\pi }{2}\right)$ and for scaling $M_{p}\left(Z\right)$.

(1) $$\left(\begin{array}{c} X_{i\;calc}\\ Y_{i\;calc}\\ Z\\ 1 \end{array}\right)=M_{p}\left(Z\right)M_{v}\left(\theta -\frac{\pi }{2}\right)\left(\begin{array}{c} x_{i}\\ y_{i}\\ z_{i}\\ 1 \end{array}\right)$$

**Figure 2:**
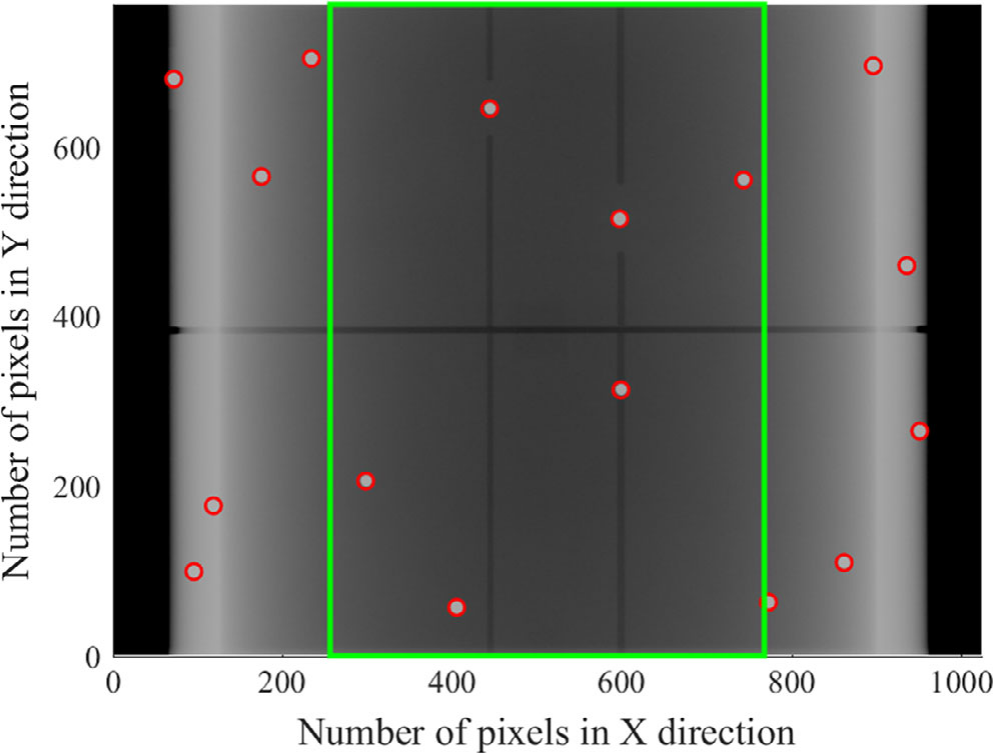
Images of data analysis to determine the gantry angle using the kV image. Red circles show 16 BBs detected on the kV image and the gantry angle was determined by the coordinates of BBs inside of the green rectangle with 512 × 768 pixels.

The image of BBs was acquired using the on‐board kV imager. After the global thresholding process, the projected coordinate $\left(X_{i},\;\;Y_{i}\;\right)$ of *i*th BB was measured. In this process, the imager sag was corrected with premeasured coordinates of the projected image from every 5 deg of gantry angle for one BB that was arranged at the isocenter.^17^ The gantry angle (θ_img_) was determined by the least squares minimization using the following formula,

(2) $$\theta_{\text{img}}=\text{arg min}\sum_{i}^{n}{\left[P_{i}-P_{i\;\text{calc}}\left(\theta \right)\right]}^{2}$$

where *n* is the number of BBs within a region of interest (ROI) that is defined as 512 × 768 pixels and placed on the center of the projected image, $P_{i}$ and $P_{i\;\text{calc}}$ are the measured and the calculated coordinates for *i*th BB. The data processing was performed using an in‐house program on MATLAB (R2017a, Mathworks, Natick, MA, USA). Figure 2 shows the image to determine the gantry angle. By calculation of θ_img_ using one BB inside the ROI, the resolution of angle per pixel ($\Delta \theta_{\text{img}}$) varies from 0.11 deg to 0.17 deg. When *n* BBs are inside the ROI, the estimated angular resolution ($\Delta {\bar{\theta }}_{\text{img}}$) can be calculated using the following equation.

(3) $$\Delta {\bar{\theta }}_{\text{img}}\;=\;\;\frac{\Delta \theta_{\text{img}}}{\sqrt{n}}$$

As shown in Fig. 2, θ_img_ is determined with images of at least six BBs, so the angular resolution can be estimated within 0.07 deg.

### 2.C Comparison in static delivery

In order to evaluate the method given in Section 2.B, θ_img_ was compared with gantry angle θ_log_ recorded in the trajectory log file (ver. 3.0, Varian Medical Systems, Palo Alto, CA, USA). The triggered kV images were acquired every 5 deg of the gantry angle through its full extent (from −180 deg to 180 deg), and θ_inc_ was measured simultaneously using a digital inclinometer (DP‐90, Niigata Seiki Co., Niigata, Japan) attached to the gantry head. θ_log_ was also compared with θ_inc_ to confirm the gantry angle only in static delivery.

### 2.D Comparison during gated arc delivery

The gantry was rotated at speeds of 2, 4 or 6 deg s^‐1^ in the clockwise (CW) or counterclockwise (CCW) direction, beam‐on time was set at 3.0 s and the interval time was set at 1.5 or 3.0 s, respectively. These conditions reproduce actual gantry rotation speeds and realistic patterns for fast and/or normal breathing in gated VMAT. The combination of maximum gantry rotation speed of 6.0 deg s^‐1^ and short interval time of 1.5 s was assumed to be the heaviest load in clinical practice. θ_img_ was determined using the image that was sampled at the moment of gate‐on or gate‐off. θ_log_ was determined using the status log of gate‐on and gate‐off that was recorded in 20 ms intervals.

## 3 RESULTS

### 3.A Comparison in static delivery

**Figure 3:**
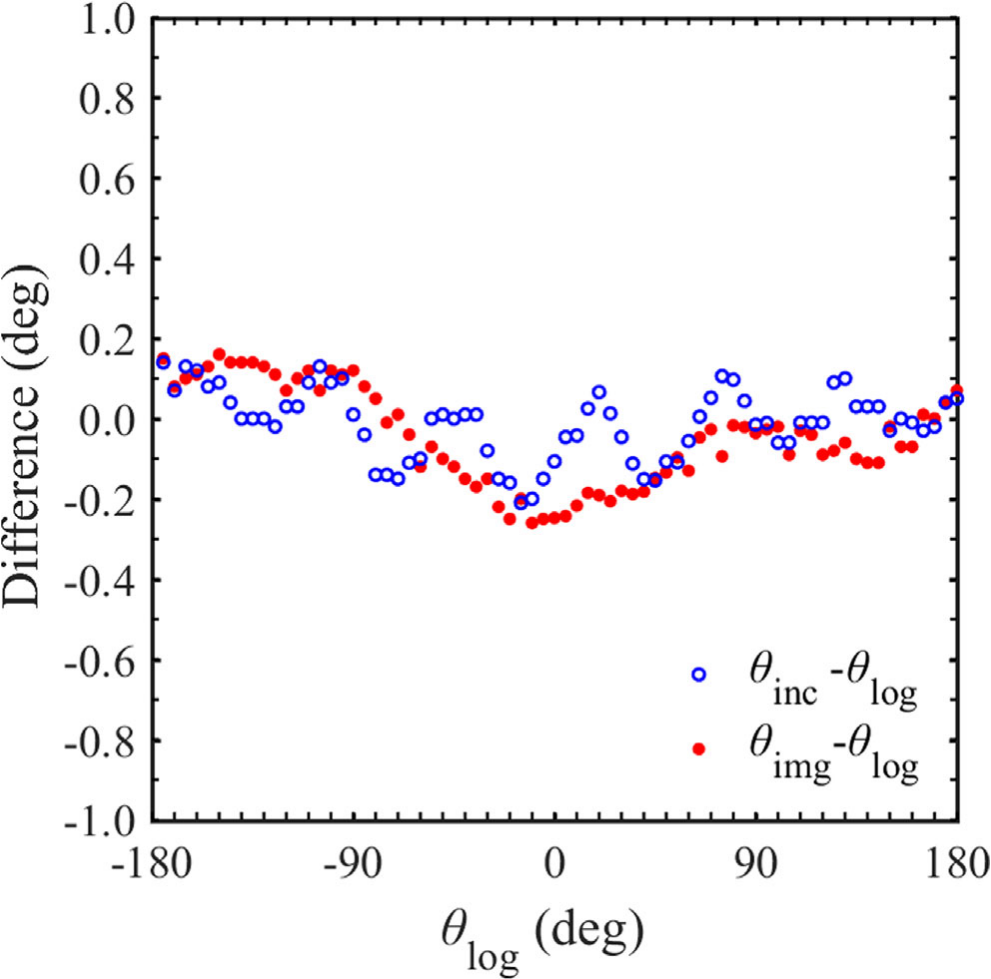
Distributions of the angular differences between θ_inc_ and θ_log_ or θ_img_ and θ_log_ in the range of the gantry angle from −180 deg to 180 deg every 5 deg.

Figure 3 shows the angular difference between θ_inc_ and θ_log_ or θ_img_ and θ_log_. In the range of a gantry angle of −180 deg to 180 deg, the mean and standard deviation (SD) of angular differences (θ_inc_ – θ_log_) was −0.01 deg ± 0.09 deg and the largest difference was 0.21 deg. These results showed good agreement, therefore θ_log_ was assumed as the gantry angle reference in this study. However, short periodicity was observed in the relationship between both methods. The DP‐90 has a resolution of 0.05 deg and precision of 0.20 deg, therefore, the periodicity may be derived from θ_inc_. θ_img_ has a high angular resolution because it is determined from images of at least six BBs. The mean and SD of angular differences (θ_img_ – θ_log_) was −0.05 deg ± 0.12 deg. The difference changed from −0.26 deg to 0.16 deg with the gantry angle during one rotation. θ_img_ had no short periodicity such as θ_inc_ and agreed with the reference angle within 0.30 deg in static delivery.

### 3.B Comparison during gated arc delivery

Table 1 shows the mean and SD of the angular difference between θ_img_ and θ_log_ at the moment of gate‐on or gate‐off. In the gate‐on mode, the angular difference was within 0.30 deg and it was independent of the gantry rotation speed and the interval time. In the gate‐off mode, the angular difference increased with the gantry rotation speed. The reason is that the gantry is stationary until triggered in the gate‐on mode, but the gantry is rotating and the image is sampled in 50 ms ± 2% after being triggered in the gate‐off mode. It was confirmed that the rotation speed was constant within 50 ms by the log analysis. Therefore, it is necessary for θ_img_ to correct 0.1, 0.2 or 0.3 deg for rotation speed 2, 4 or 6 deg s^‐1^, respectively. After the correction, the mean of the angular difference was within ±0.10 deg and it became independent of the gantry rotation speed in the gate‐off mode.

**Table 1:** The mean and SD of the difference between θ_img_ and θ_log_ at the moment of gate‐on and gate‐off during gated arc delivery. In the case of the gate‐off moment, θ_img_ was corrected 0.1, 0.2 or 0.3 deg for rotation speed 2, 4 or 6 deg s^-1^, respectively.

Rotation speed (deg s^-1^)	Interval time (s)	Angular difference during CW rotation (deg)			Angular difference during CCW rotation (deg)		
		Gate‐on	Gate‐off		Gate‐on	Gate‐off	
			w/o imaging latency correction	w/ imaging latency correction		w/o imaging latency correction	w/ imaging latency correction
2.0	1.5	0.04 ± 0.12	0.17 ± 0.12	0.07 ± 0.12	0.01 ± 0.12	−0.12 ± 0.13	−0.02 ± 0.13
4.0		0.04 ± 0.12	0.29 ± 0.13	0.09 ± 0.13	0.02 ± 0.11	−0.22 ± 0. 15	−0.02 ± 0.15
6.0		0.07 ± 0.10	0.39 ± 0.17	0.09 ± 0.17	0.00 ± 0.13	−0.34 ± 0.16	−0.04 ± 0.16
2.0	3.0	0.04 ± 0.12	0.18 ± 0.12	0.08 ± 0.12	0.02 ± 0.13	−0.13 ± 0.14	−0.03 ± 0.14
4.0		0.05 ± 0.11	0.29 ± 0.14	0.09 ± 0.14	0.01 ± 0.13	−0.22 ± 0.15	−0.02 ± 0.15
6.0		0.04 ± 0.12	0.38 ± 0.18	0.08 ± 0.18	0.02 ± 0.14	−0.35 ± 0.18	−0.05 ± 0.18

## 4 DISCUSSION

To evaluate the gantry angle during respiratory‐gated VMAT, a new measurement‐based QA was proposed. The gantry angle was determined with specific positions of BBs on the triggered kV image. The IsoCal phantom is suitable for the proposed method because the BBs are arranged exactly in it. Other phantoms in which the BB arrangement is known may also be adapted to the proposed method. In the previous study, the gantry angle was evaluated using radiochromic film or a cine electronic portal imaging device (cine‐EPID) image.^14^, ^18^, ^19^ Radiochromic film is a low sensitivity device so that a slow rotation speed was required for sampling.^14^ The cine‐EPID has large time uncertainty due to a slower sampling rate of 2.5 Hz and the initial EPID frame will be lost.^18^ Consequently, this makes it difficult to evaluate the gantry angle at a given moment such as gate‐on or gate‐off. On the other hand, the triggered kV image can be acquired with high sensitivity and short sampling time. Therefore, the gantry angle is sampled synchronously with gate‐on or gate‐off moment at maximum gantry rotation speed. Furthermore, the proposed method can be applied to VMAT plans because the kV image is independent of the treatment beam.

**Figure 4:**
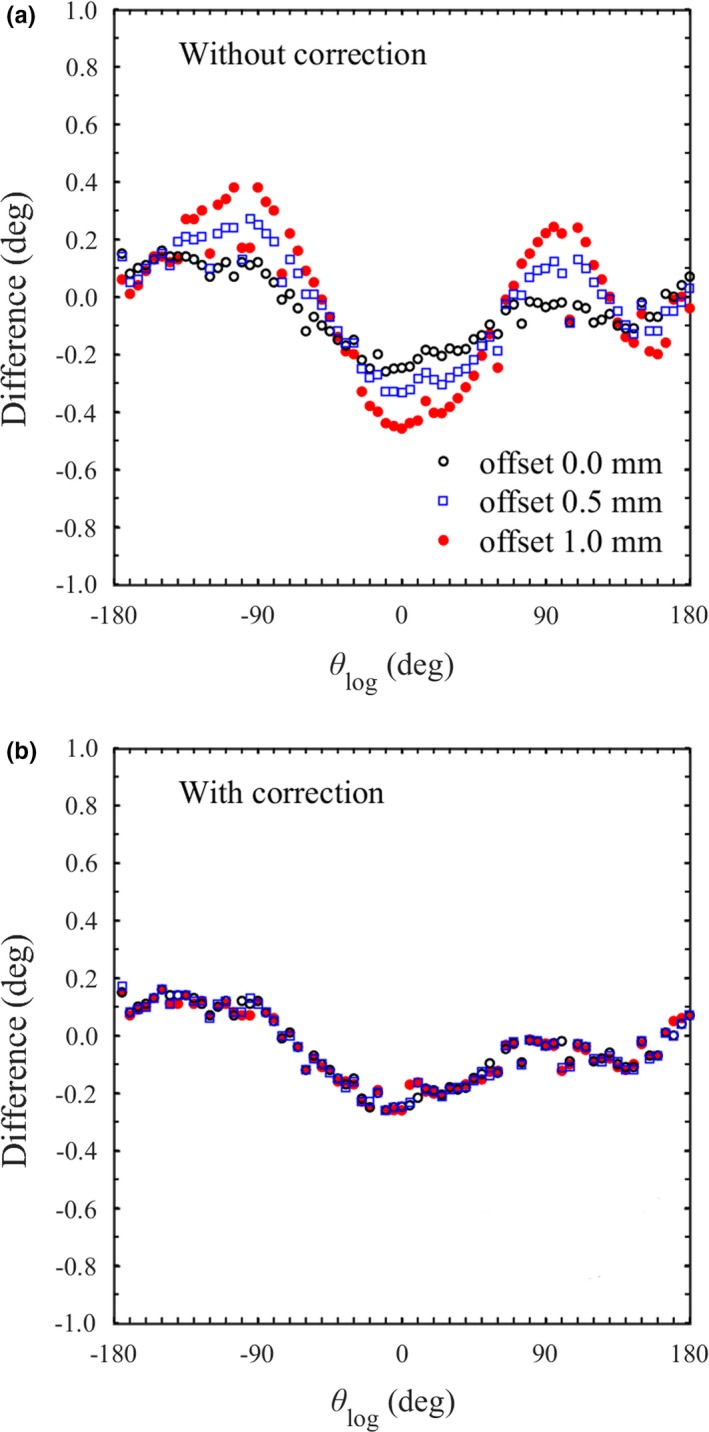
For each phantom offset (0.0, 0.5 and 1.0 mm for each axis), the differences between θ_img_ and θ_log_ at several gantry angles (a) without and (b) with the phantom offset correction.

In static delivery, the angular difference between the proposed method and log analysis was varied slightly during one rotation. It seems that the long periodicity variation is caused by the gantry miscalibration or sagging, which cannot be detected by the trajectory log. The short periodicity variation is caused by the pixel size, phantom manufacturing and phantom setting. The pixel size of the triggered kV image is 0.39 mm, and BBs are arranged at a specified position with an uncertainty of ±0.5 mm. The phantom setting can be slightly displaced from the isocenter within 1.0 mm due to misalignment of the in‐room lasers and inter‐operator variability. Figure 4(a) shows the difference between θ_img_ and θ_log_ when the phantom was set with an offset of 0.0, 0.5 or 1.0 mm from the isocenter. The mean and SD of the difference was −0.05 deg ± 0.12 deg, −0.02 deg ± 0.17 deg and −0.02 deg ± 0.24 deg when the offset is 0.0, 0.5 and 1.0 mm, respectively. As the offset increased, the angular differences became larger.

To evaluate the gantry angle accurately, the phantom offset $\left(x_{\text{T}},y_{\text{T}},z_{\text{T}}\right)$ must be determined. First, the average of the positional differences between the measured and the calculated coordinates for all BBs $\delta \bar{X}$ and $\delta \bar{Y}$ are calculated using kV images sampled at representative gantry angles (from −180 deg to 180 deg every 30 deg). $\delta \bar{X}$ can be fit as a simple periodic function of the gantry angle that included the sine component induced by the lateral displacement *x*_T_, and the cosine component induced by the vertical displacement *z*_T_. The probable offset $x_{\text{T}}^{*}$ and $z_{\text{T}}^{*}$ can be determined using the following equation,

(4) $$\left(x_{\text{T}}^{*},z_{\text{T}}^{*}\right)=\text{arg min}\sum_{k=\;1}^{m}{\left\{\alpha \delta \bar{X}\left(\theta_{k}\right)-\left[x\sin\left(\theta_{k}\right)+z\cos\left(\theta_{k}\right)\right]\right\}}^{2}$$

where *m* is the number of representative gantry angles, and *α* is the ratio of source‐axis distance (SAD) to SDD. In contrast, $\delta \bar{Y}$ is affected by only the longitudinal displacement *y*_T_. Therefore, the probable offset $y_{\text{T}}^{*}$ is determined using the following equation,

(5) $$y_{\text{T}}^{*}=\frac{1}{m}\sum_{k=1}^{m}\alpha \delta \bar{Y}\left(\theta_{k}\right)$$

For given phantom settings, $\left(x_{\text{T}}^{*},y_{\text{T}}^{*},z_{\text{T}}^{*}\right)$ can be quantified <0.1 mm. The coordinates of *i*th BBs (*x_i_*, *y_i_*, *z_i_*) in the reference coordinate system are transformed by the matrix $M_{t}\left(x_{\text{T}}^{*},y_{\text{T}}^{*},z_{\text{T}}^{*}\right)$ and Eq. (1),

(6) $$\left(\begin{array}{c} X_{i\;\text{calc}}\\ Y_{i\;\text{calc}}\\ Z\\ 1 \end{array}\right)=M_{p}\left(Z\right)M_{v}\left(\theta -\frac{\pi }{2}\right)M_{t}\left(x_{\text{T}}^{*},y_{\text{T}}^{*},z_{\text{T}}^{*}\right)\left(\begin{array}{c} x_{i}\\ y_{i}\\ z_{i}\\ 1 \end{array}\right)$$

The gantry angle is determined using Eq. (2). Figure 4(b) shows the difference between θ_img_ and θ_log_ after the phantom offset correction. The mean and SD of the difference was decreased to −0.05 deg ± 0.12 deg even though the phantom offset is 0.5 or 1.0 mm. This result suggests that the gantry angle can be evaluated independently from the phantom setting by the proposed method. The correction for phantom rotation has not been implemented yet, so the phantom rotation was adjusted with a six degrees of freedom couch in this study.

**Figure 5:**
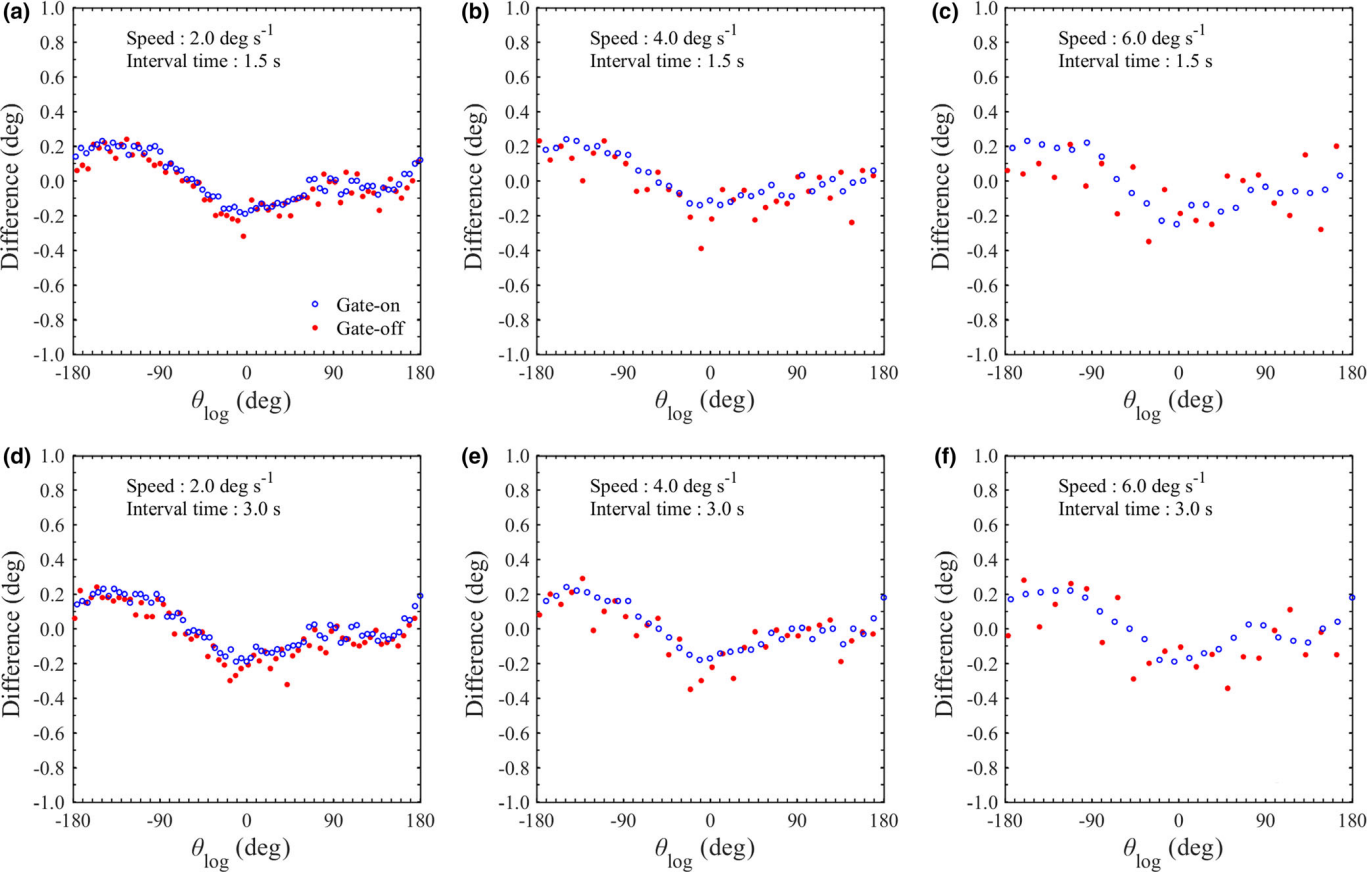
Distributions of the difference between θ_img_ with imaging latency correction and θ_log_ at the moment of gate‐on and gate‐off during gated arc delivery. The gantry was rotated at speeds of 2.0, 4.0 or 6.0 deg s^-1^ in the counterclockwise direction with gating intervals (a–c) 1.5 s or (d–f) 3.0 s, respectively.

In previous studies evaluating the machine performance of gated VMAT, several authors analyzed the difference between the planned and the recorded gantry angle using the machine log.^9^, ^10^ However, the machine log records the output of the encoder, so that the uncertainty due to the miscalibration and mechanical sagging will be disregarded. Moreover, the log is recorded discretely with the same sampling interval, so it has a discrepancy depending on the gantry rotation speed. These uncertainties of log analysis were not discussed in previous reports, so they should be confirmed with the measurement‐based QA. Hubley et al. reported on measurement‐based QA using radiochromic film, however, it could not be used to evaluate an angular difference <0.5 deg and could not be applied at rotational speeds exceeding 2.3 deg s^‐1^.^14^ The gantry rotation speed in gated VMAT varies rapidly from slower to its maximum of 6.0 deg s^‐1^, therefore, a more sensitive QA device is required. The proposed method is able to determine the gantry angle at the moment of gate‐on or gate‐off in arc delivery with a reasonable angular resolution. In addition, this method can detect miscalibration and mechanical sagging because BBs are exactly arranged at specific positions in the phantom. As shown in Fig. 5, the gantry angles determined using the proposed method agreed well with the results of the log analysis. The trajectory log is recorded at intervals of 20 ms, so the mean of angular difference between both methods was within ±0.10 deg and the largest difference was 0.41 deg even though the gating condition was the heaviest load (the combination of a maximum gantry rotation speed of 6.0 deg s^‐1^ and short interval time of 1.5 s). Therefore, the angular difference will not exceed the result of the heaviest load even in other gating conditions. The proposed method will become important for detecting mechanical issues due to repeated gantry stops and restarts in gated VMAT even if patient‐specific dose verification is replaced by log analysis.

## 5 CONCLUSIONS

Several authors have evaluated the difference between the planned and the recorded gantry angle by log analysis for respiratory‐gated VMAT. However, log analysis detects only the output of the encoder so that miscalibration and mechanical sagging will be disregarded. Measurement‐based QA of the gantry angle using the kV imaging system and cylindrical phantom with 16 BBs was proposed and it was compared with log analysis in this report. The proposed method acquired images with a short sampling time; consequently, the gantry angle can be sampled synchronously with gate‐on or gate‐off moment at maximum gantry rotation speed. The proposed method will become important for detecting mechanical issues due to repeated gantry stops and restarts in gated VMAT even if patient‐specific dose verification is replaced by log analysis.

## CONFLICT OF INTEREST

We have no conflict of interest to declare.
